# Guidance on the use of complex systems models for economic evaluations of public health interventions

**DOI:** 10.1002/hec.4681

**Published:** 2023-04-20

**Authors:** Penny R. Breeze, Hazel Squires, Kate Ennis, Petra Meier, Kate Hayes, Nik Lomax, Alan Shiell, Frank Kee, Frank de Vocht, Martin O’Flaherty, Nigel Gilbert, Robin Purshouse, Stewart Robinson, Peter J Dodd, Mark Strong, Suzy Paisley, Richard Smith, Andrew Briggs, Lion Shahab, Jo‐An Occhipinti, Kenny Lawson, Thomas Bayley, Robert Smith, Jennifer Boyd, Visakan Kadirkamanathan, Richard Cookson, Monica Hernandez‐Alava, Christopher H. Jackson, Amanda Karapici, Franco Sassi, Peter Scarborough, Uwe Siebert, Eric Silverman, Luke Vale, Cathal Walsh, Alan Brennan

**Affiliations:** ^1^ School of Health and Related Research University of Sheffield Sheffield UK; ^2^ British Medical Journal Technology Appraisal Group London UK; ^3^ MRC/CSO Social and Public Health Sciences Unit University of Glasgow Scotland UK; ^4^ School of Geography University of Leeds Leeds UK; ^5^ Department of Public Health LaTrobe University Melbourne Australia; ^6^ Centre for Public Health Queen's University Belfast Belfast UK; ^7^ Population Health Sciences Bristol Medical School University of Bristol Bristol UK; ^8^ NIHR Applied Research Collaboration West (ARC West) Bristol UK; ^9^ Department of Public Health, Policy and Systems University of Liverpool Liverpool UK; ^10^ CRESS University of Surrey Guildford UK; ^11^ Department of Automatic Control and Systems Engineering University of Sheffield Sheffield UK; ^12^ Business School University of Newcastle Newcastle UK; ^13^ Lumanity‐HEOR South Yorkshire Sheffield UK; ^14^ College of Medicine and Health University of Exeter Exeter UK; ^15^ London School of Hygiene & Tropical Medicine London UK; ^16^ Department of Behavioural Science and Health UCL London UK; ^17^ Brain and Mind Centre University of Sydney New South Wales Camperdown Australia; ^18^ United Kingdom Health Security Agency Birmingham UK; ^19^ MRC/CSO Social and Public Health Sciences Unit University of Glasgow Glasgow UK; ^20^ Centre for Health Economics University of York Heslington UK; ^21^ MRC Biostatistics Unit University of Cambridge Cambridge UK; ^22^ NIHR SPHR London School of Hygiene and Tropical Medicine London UK; ^23^ Centre for Health Economics & Policy Innovation Imperial College Business School London UK; ^24^ Nuffield Department of Population Health University of Oxford Oxfordshire Oxford UK; ^25^ Department of Public Health, Health Services Research and Health Technology Assessment UMIT TIROL ‐ University for Health Sciences and Technology Hall in Tirol Tyrol Austria; ^26^ Division of Health Technology Assessment and Bioinformatics ONCOTYROL ‐ Center for Personalized Cancer Medicine Innsbruck Austria; ^27^ Center for Health Decision Science Departments of Epidemiology and Health Policy & Management Harvard T.H. Chan School of Public Health Massachusetts Boston USA; ^28^ Program on Cardiovascular Research, Institute for Technology Assessment and Department of Radiology Massachusetts General Hospital Harvard Medical School Massachusetts Boston USA; ^29^ Health Economics Group Population Health Sciences Institute Newcastle University Newcastle UK; ^30^ Health Research Institute and MACSI University of Limerick Limerick Ireland

**Keywords:** complex systems, economic modeling, public health

## Abstract

To help health economic modelers respond to demands for greater use of complex systems models in public health. To propose identifiable features of such models and support researchers to plan public health modeling projects using these models. A working group of experts in complex systems modeling and economic evaluation was brought together to develop and jointly write guidance for the use of complex systems models for health economic analysis. The content of workshops was informed by a scoping review. A public health complex systems model for economic evaluation is defined as a quantitative, dynamic, non‐linear model that incorporates feedback and interactions among model elements, in order to capture emergent outcomes and estimate health, economic and potentially other consequences to inform public policies. The guidance covers: when complex systems modeling is needed; principles for designing a complex systems model; and how to choose an appropriate modeling technique. This paper provides a definition to identify and characterize complex systems models for economic evaluations and proposes guidance on key aspects of the process for health economics analysis. This document will support the development of complex systems models, with impact on public health systems policy and decision making.

## 1 INTRODUCTION

There have been calls for greater use of complex systems methods to inform public health decision making (Diez Roux, 2011; Lich et al., 2013; Rutter et al., 2017). Computational models can be useful to evaluate public health interventions operating within complex systems, however, there are few examples of economic evaluations employing complex systems models (Shiell et al., 2008; Squires & Boyd, 2019).

### 1.1 What is a computational model?

A computational model describes a simplified representation of reality in which a system is described using mathematical relationships (Caro et al., 2012). Such models combine evidence to quantify the future performance of parts of the system and address questions that are difficult to answer using primary empirical research approaches (Brennan et al., 2006). Within public health this includes: planning services, supporting infectious disease surveillance, policy impact analysis, economic evaluation, testing determinants of disease patterns, investigating disease trajectories, and testing intervention scenarios (Briggs et al., 2016). Models can characterize the population at the individual‐level, in which people are distinct units, or at the aggregate level, using population averages.

### 1.2 Features of a complex system

A system refers to problem situations characterized by having interconnected elements (Meadows, 2008), with multiple causes and consequences (Chapman, 2004). System complexity increases with the intricacy of the relationships between elements (Rickles et al., 2007). Therefore, it is not the number of interactions that is the defining characteristic of dynamic complexity, but rather the nature of interactions and their generation of emergent outcomes (Holland, 2014). Emergent outcomes are properties, perhaps observed at an aggregate‐level of a complex system, that cannot be predicted by considering the elements within it in isolation, and are more than just the sum of its parts. Thus, in complex system problems, the effects of any single intervention cannot be determined in isolation. Each decision that is made depends on others, multiplying the counterfactuals that need to be considered (Ornstein et al., 2020).

Public health challenges can be conceptualized as complex systems problems because they involve: (i) mutual interdependencies between elements of the system, where effect directions, sizes, accumulation and timings are not well‐understood or captured by research methods grounded in linear models of cause and effect; (ii) actors who have diverse sets of priorities, values and understanding of the problem; (iii) costs, benefits and harms spread across many parts of the system; and (iv) deep uncertainties due to rapidly shifting geo‐political and economic contexts potentially changing population, demographic and/or behavioral dynamics and the interplay between social determinants and service system factors that influence the impacts of interventions (Meier et al., 2019).

Obesity provides a public health example of a systems problem in which interplay between numerous biological, environmental, social, political and economic factors influences obesity, which in turn can have implications for policy evaluation. A model describing a simple pathway between disease trajectory and health outcomes will be sufficient if the intervention produces consistent effects regardless of context. However, if the intervention interacts with other factors affecting the evolution, and consequences of the disease, this modeling approach will overlook emergent outcomes.

### 1.3 Economic modeling in public health

Economic evaluation is a core component of all phases of intervention research in public health (Skivington et al., 2021). Economic evaluation using modeling techniques can estimate the value of public health investments, exploring incremental and population effects of changes in policies (Squires & Boyd, 2019). Modeling approaches used in economic evaluation often develop from the methods of health technology assessment, that is, decision‐trees and Markov models, in which the implementation context is not explicitly modeled (Lawson et al., 2022). Discrete Event Simulation has been recommended to extend model complexity in economic evaluations (Karnon & Haji Ali Afzali, 2014), whereas in the public health literature complex systems models are often identified by other model traditions, such as system dynamics or agent‐based models (ABM)s (Atkinson et al., 2015; Bicket et al., 2020; Carey et al., 2015; McGill et al., 2021; Xue et al., 2018). Guidance is needed to overcome barriers in translating cross‐disciplinary knowledge given the breadth of the systems science literature (Trochim et al., 2006).

### 1.4 Complex systems modeling in public health

Complex system methods for public health have been discussed extensively in the literature (Leischow & Milstein, 2006; Lich et al., 2013; Luke & Stamatakis, 2012; Rutter et al., 2017; Tracy et al., 2018; Trochim et al., 2006). However, several reviews highlight the competing definitions of complex systems (Leischow & Milstein, 2006; Lich et al., 2013; Trochim et al., 2006). As such, it is useful to specify the distinguishing features of a Complex System Model (CSM) that can be identified from the model structure and mathematical relationships, rather than focusing on modeling traditions for example, Systems Dynamics and agent‐based models. A definition of a CSM for public health economic modelers will enable consistent labeling of models, limit conceptual stretching, a more efficient description of methods, and, where appropriate, encourage the adoption of CSM.

### 1.5 Aims of this guidance

The aims of this manuscript are to (i) propose a definition of computational CSM that highlights the critical modeling features for economic modelers to use in public health models (Section A), (ii) support economic modelers to identify situations when a CSM is needed (Section B), (iii) identify appropriate modeling types for economic modelers to choose from (Section C), (iv) highlight useful approaches/methods in CSM (Section D).

## 2 METHOD

The guidance was written in consultation with an international consortium of academic experts in CSM and related fields. In September 2020, 44 academic experts from the project leader's network and identified using a snowball approach to recruitment were invited to attend two workshops (October 2020 and March 2021). Participants included established academics and researchers along with PhD students, to provide an understanding of the challenges involved in modeling in public health. Of these, 42 attended the workshops and 36 contributed to the manuscript.

The aims and scope of the first workshop were informed by an organizing committee (PB, AB, HS, KE) and a scoping review of the literature on CSMs in public health (Table S1 in the supplementary material). The scoping review aimed to identify examples of CSM and catalog the various modeling methods used (Table S2 in the supplementary material). The review used and adapted a previous systematic review of complex systems methods (Carey et al., 2015).

Discussions from the two workshops provided direction on the scope, structure, and content of this guidance document. Details of the review and workshops are described in Appendix A and Appendix B of the supplementary material.

We tested the application of the definition of complex systems models against a set of CSM case studies. The case studies were identified by either the literature review or workshop participants to represent different model types, including but not limited to economic studies, across a range of public health topics. This process identified eight public health CSMs. We systematically cataloged methods used in the development of these models with input from workshop participants.

## 3 SECTION A: DEFINITION OF COMPLEX SYSTEMS MODELS

A public health oriented CSM is a quantitative, dynamic, non‐linear model that incorporates feedback, and interactions among model elements, in order to capture emergent outcomes and estimate health, economic and potentially other consequences to inform public policies.

To aid with the interpretation of the definition and guidance, we provide a glossary of terms in Table S3 of the supplementary material. The definition recognizes four overlapping critical features: dynamic, non‐linear, feedback and interaction that can be programmed into an economic model that in combination give rise to the properties of a complex system. A figure illustrating the critical features is provided in Figure S1 of the supplementary material. A model incorporating the critical features will add complexity and predispose the model to complex properties. In contrast, the quantity of elements, or the intricacy of the intervention (Shiell et al., 2008), may make the model complicated, but not necessarily complex. Complexity is not the same thing as complication: non‐complex models can be complicated, and complex models can be (relatively) simple. There are numerous examples of non‐complex, but complicated, system models informing public health decisions linking public health policies to a broad range of outcomes (Springmann et al., 2016; Holmes, J et al., 2014; Thomas et al., 2022). These models do not include the critical features, that is, not dynamic (Springmann et al., 2016), do not include interactions (Holmes et al., 2014; Springmann et al., 2016), and do not include feedback loops (Holmes et al., 2014; Springmann et al., 2016; Thomas et al., 2022).

High system complexity is characterized by several properties: feedback, adaptation, emergent outcomes and non‐linearity. These properties can be programmed into a CSM if it has the critical features so that the changes that ripple through a system (i.e., an intervention) are non‐linear (not proportional to the size of the initial stimulus), adaptive and lead to emergent outcomes. In a CSM model changes to one element cause dynamic changes in other parts of the model, which continue to feedback around the model amplifying or dampening the initial change and resulting in further changes to the initial element. The ramifications of these relationships will be greater with more critical features connecting model elements in the system.

Table 1 describes how the four features of complexity were demonstrated in eight exemplar public health CSMs (Brailsford et al., 2012; Dodd et al., 2010; Keogh‐Brown et al., 2019; Occhipinti et al., 2021; Probst et al., 2020; Stankov et al., 2019; Tobias et al., 2010; Viana et al., 2014).

**TABLE 1:** Eight selected Public Health Complex System Model (CSM) case studies that demonstrate the key features and insights of the approach.

Authors	Aims	Study description	Why it counts as complex systems model	What useful insights did the CSM provide?
Dodd et al., 2010	To explore the effect of human immunodeficiency virus (HIV) transmission epidemiology on the impact of universal test‐and‐treat interventions.	**Partial differential equations.** The model includes infectiousness that changes over time since HIV infection; under antiretroviral therapy infectiousness is reduced and life‐expectancy extended.	**Non‐linear:** The model is a non‐linear dynamical system.	The impact of a universal test‐and‐treat intervention was shown, for matched prevalence, to depend on heterogeneity and mixing of contacts. In some situations, less aggressive interventions achieved the same results, whereas in others, reductions were lower; annual strategies were not necessarily the most cost‐efficient. The potential for incomplete implementation or coverage to increase long‐term antiretroviral therapy (ART) costs was demonstrated.
			**Dynamic:** The model accounts for the population dynamics of HIV infection over time.	
			**Interactions**: The PDEs model assortative mixing between high‐ and low‐risk segments of a heterosexual population.	
			**Feedback:** There is positive feedback ‐ higher infection prevalence drives higher incidence of infection.	
Brailsford et al., 2012	To evaluate the costs and benefits of alternative breast cancer screening policies in a screening model that incorporates human behavior.	**Discrete event simulation.** A three‐phase discrete event simulation was built to model breast cancer and screening policies and extended to include patients' behavioral characteristics.	**Non‐linearity:** Non‐linear model specifications for tumor growth were simulated to allow for time dependency in tumor growth.	The model enables a broad range of experimental settings to observe the impact of screening strategies and health behaviors on health outcomes. The method for modeling behavior did not substantially alter the model outcomes. However, incorporating theory‐led models of human behavior allow decision makers to design public health interventions that increase the likelihood of attendance. The frequency of screening impacted future participation, and in this scenario the policy more effective.
			**Dynamic:** Individuals are simulated from birth until death and the timing of screening is an important parameter in policy evaluation.	
			**Interaction:** Interaction between the individual and the screening service is determined by the timing of screening, and the actions by the individual to attend screening.	
			**Feedback:** The model includes theories of human behavior to inform attendance at screening. Adaptive theories in which previous attendance at screening impacts the likelihood of future attendance were modeled.	
Probst et al., 2020	Aims: To develop a theoretical framework to explain macro‐level trends in drinking and test the effect of policies on alcohol consumption.	**Agent‐based model.** An individual‐level model was developed to simulate dynamic normative mechanisms and behavioral rules underlying drinking behavior over time. The model encompassed drinking norms and their impact on frequency and quantity of alcohol use. Three experiments were performed to test the modeled normative mechanisms.	**Non‐linearity:** Changes made to the input parameters in the model for the three experiments did not produce proportional changes in drinking behavior.	The model allowed the researchers to examine the degree that individual‐level mechanisms could explain more macro‐level phenomena in drinking behavior. Three experimental scenarios were programmed to observe the effectiveness of policies on drinking trends. An increase in the desire to drink led to the most meaningful changes in the population's drinking behavior indicating the high levels of autonomy in decisions to drink. A higher degree of “receptiveness” toward normative influence can be considered a prerequisite to behavioral changes.
			**Dynamic:** The model simulates micro‐level decisions to drink and changes in dynamic social‐level norms to observe macro‐level trends in alcohol consumption over time.	
			**Interactions:** The individual interacts with the environment through the social norms that influence the likelihood that they and other individuals in the model would drink. Therefore, individual decisions to drink modify the macro‐level social norms.	
			**Feedback:** Two feedback loops were programmed to adjust injunctive norms (perceived acceptability) over time in response to perceived harm to society or prevalence of drinking in their age/gender reference group. A further feedback loop adjusts descriptive norms over time (perceptions of drinking by people in their age/gender reference group).	
Tobias et al. (2010).	To compare the impact of different smoking cessation services in New Zealand on smoking prevalence, tobacco consumption, and tobacco‐attributable mortality. To provide a decision tool to support the design and evaluation of tobacco control policies.	**Systems dynamics.** The model has six components. The population component describes the flow of people between smoking states (‘never‐ smokers’, ‘current‐ smokers’, ‘ex‐smokers’ etc.), conditional on rates of initiating, quitting, and mortality among smokers and ex‐smokers. These rates are affected by role modeling and household composition. Other components describe smoking prevalence; tobacco consumption; second‐ hand smoke; relative risks; and tobacco attributable deaths as a result of smoking or exposure to second‐hand smoke.	**Non‐linearity:** Non‐linear behavior occurs as a result of feedback loops.	The model enables the evaluation of a range of policies that aim to prevent tobacco‐related harm. The authors tested the effects of an intervention package that acts through a range of channels from price changes, marketing, and service provision. They estimated that this package could reduce tobacco‐related mortality by 11% within 35 years. This information informed the decision in 2007 to increase funding for smoking cessation interventions by NZ$42 million. The same model in a previous study was used to test the effect of hypothetical cigarette modifications (i.e. manufacturing either less toxic or less addictive cigarettes) on smoking prevalence and harm. They found that these policies would lead to a degree of compensatory smoking and the possible expansion of the tobacco market, and so could only be helpful in combination with regulations like marketing bans and tax increases.
			**Dynamic:** The model can be simulated for 50 years, with the emphasis on the first 20–30. Variables can change over time. The model produces yearly estimates of smoking prevalence & harm, reflecting the dynamic effects of interventions.	
			**Interactions:** The six components of the model interact and produce model outcomes. For example, the tobacco consumption component interacts with the relative risk component, which affects the mortality component and the size of the smoking population, which affects the exposure to second‐hand smoke and so on.	
			**Feedback:** Peer smoking and parental smoking both create reinforcing feedback loops that increase the number of youth smoking initiations and the persistence of smoking in adulthood through role modeling effects.	
Occhipinti, A et al., 2021	The study aimed to i) identify the likely impact over time of mental health and suicide prevention interventions (ii) determine the value and balance of investments across the social determinants of mental health in the region, and (iii) determine the best combination of strategies to deliver the greatest impacts on suicidal behavior.	**Systems dynamics.** The model is separated into several components. The population component describes the characteristics of the population. The population moves between states of psychological distress conditional on social determinants of psychological distress components, including adverse early life exposures, homelessness, employment, domestic violence, and substance abuse. A mental health service component represented the pathway of care for psychological distress. Suicidal behavior was a key outcome.	**Dynamic:** The model reported dynamic changes in model outputs over 40 years.	The model provided decision makers and stakeholders with a tool to investigate alternative scenarios related to the timing of implementation of interventions, their scale and intensity, and to test alternative assumptions regarding level of intervention uptake to inform strategic decision making. Initiatives to improve social connectedness were the most effective. The model demonstrated that the greatest impacts on suicidal behavior are observed when mental health and suicidal initiatives are combined with interventions to address key social determinants. Adding all mental health initiatives was only marginally better than providing a targeted combination. This suggests that there are diminishing returns from investing additional investing in programs and initiatives beyond the best combination, and complex systems models can assist by prioritizing services when resources are limited.
			**Feedback:** Bi‐directional relationships between model components lead to unpredictable dynamic changes in outputs. Bidirectional relationships are observed between social determinants, social determinants and psychological distress, and psychological distress and the mental health system.	
			**Non‐linearity:** Non‐linearity is assumed due to structures and connections between elements in the system.	
			**Interactions:** Interactions between model components affect the dynamic relationships in the model. For example, the flow through the mental health care system is impacted by health care capacity, which is affected by rates of psychological distress and suicidal behavior.	
Keogh Brown (2019)	To generate an integrated quantification of the combined macroeconomic, disease and population burden of palm cooking oil consumption in a major palm oil consuming country context, Thailand.	**Macroeconomic model** The model describes a Computable General Equilibrium framework (CGE) sectoral mathematical model of the whole economy to describe productive labor supply, consumption and savings, government taxation and trade. The model includes a non‐economic sub‐model to simulate changes in household nutritional intake on health, health spending, and labor supply. This feeds back into the macroeconomic model to generate a new equilibrium.	**Dynamic**: The simulations are run over a 20 years time horizon.	The model estimated the health‐related economic, disease and population burden of palm cooking oil consumption in Thailand. The multi‐sector CGE model comprehensively captured economic spill‐overs, interactions and wage effects to value productive labor. The model allowed the full integration of a health sub‐model to capture transmission between consumption of food commodities and health outcomes. The feedback between health and the economy could be captured in the model. The model provides a full valuation of the macroeconomic impacts of the policies and can estimate these impacts by sectors to observe how the impacts are distributed across the economy.
			**Feedback:** The model captures feedback from the macroeconomy to household consumption, health, and the population. Changes in palm oil consumption lead to changes in health, which impacts the demographic profile, labor market supply, income, savings and further modifies consumption behavior in the population.	
			**Non‐linearity**: The disease burden model accounts for interactions and feedback effects between health, the macro‐economy and the population. These feedback loops create non‐linear relationships between the policies and model outcomes.	
			**Interactions:** The CGE framework captures interactions between economic sectors.	
Stankov et al. (2019)	To explore factors that influence the prevalence of alcohol misuse and depression in adults and to investigate the impact of tax policies and social connectedness interventions on the prevalence of depression and alcohol misuse.	**Agent‐based model.** The model included 540 agents representing older adults (65 years old or above) and the alcohol outlets in their neighbourhoods. Agents' drinking status was assigned in each time step based on a combination of individual, social and environmental factors. Individual factors included affinity for excessive consumption and depression, social factors included alcohol consumption of neighbors and cohabitants, and environmental factors alcohol pricing and access to retailers. Depression was influenced by past predisposition to depression, social connectedness, and affinity toward excessive drinking.	**Dynamic:** Individuals are simulated over a 5‐year period. 260 time steps were used, with each time step covering a week of real time. An individual's characteristics such as risk of depression, risk of excessive drinking change over time.	The model was able to adequately reproduce the prevalence of depression and alcohol misuse found in the real‐world data. The model calibration suggested that alcohol misuse and depression were related bi‐directionally, but the size of the best fit parameters were quite small suggesting that the feedback effect might only be slight. The model also provides quantitative evidence on the plausible effects of various interventions, specifically the effect of increasing taxation on alcohol and the effect of increasing social connectedness on depression and alcohol consumption. Within the model, tax interventions resulted in lower alcohol consumption, but had a minimal impact on depression, whilst social connectedness interventions reduced the prevalence of depression without substantially impacting alcohol misuse. Combinations of the interventions did not impact depression or alcohol misuse more than each intervention alone. This further suggested that feedback processes within the model were relatively weak.
			**Feedback:** Feedback exists in the model between individuals' likelihood of excessive drinking and their likelihood of being depressed.	
			**Non‐linearity:** Non‐linearity exists in the model through feedback loops, as well as through effects from the agents' personal networks. Individuals both affect and are affected by the drinking status of those in their personal network.	
			**Interactions:** Agents interact with both other agents and their environment. The characteristics of the other agents within this personal network, and the characteristics of the local environment around their residence influence the agents' likelihood of being depressed and drinking excessively.	
Viana et al., 2014	To understand how *chlamydia* screening and service provision impact interact to reduce overall disease incidence	**Hybrid models.** A systems dynamic model generates the monthly demand for Chlamydia services to input into a discrete event simulation. The discrete event simulation exports the treated and untreated populations back to the systems dynamics model.	**Dynamic:** The two models would produce data to input into each model in monthly intervals.	Each individual model produced interesting insights. In the DES model staffing levels can be altered. In the SD screening strategies were tested. When interventions were combined the models illustrated that poor performance at the Chlamydia clinic could lead to higher rate of infections in the community because of the bad reputation (long waiting times) from the clinic. The model can support decision making for community screening, or within the clinic, to observe how the changes impact on the other aspects of the system and will feedback to the primary system of interest.
			**Feedback:** In the systems dynamics model there are feedback loops to determine the susceptible and infected populations. These are impacted by the proportion of patients with a full recovery and one representing recovery with sequelae.	
			**Non‐linearity:** In the DES waiting times influence the proportion of untreated patients. In the SD model Chlamydia infections were conditional on many dynamic parameters including feedback and interactions with the DES model.	
			**Interactions:** The aggregate population prevalence and detected levels of Chlamydia interact with the DES model, to produce estimates of transitions between high and low risk groups and update the level of infection in the population.	

Abbreviation: DES, Discrete Event Simulation.

## 4 SECTION B: WHEN SHOULD, AND SHOULDN'T, COMPLEX SYSTEM MODELS BE USED?

**FIGURE 1:**
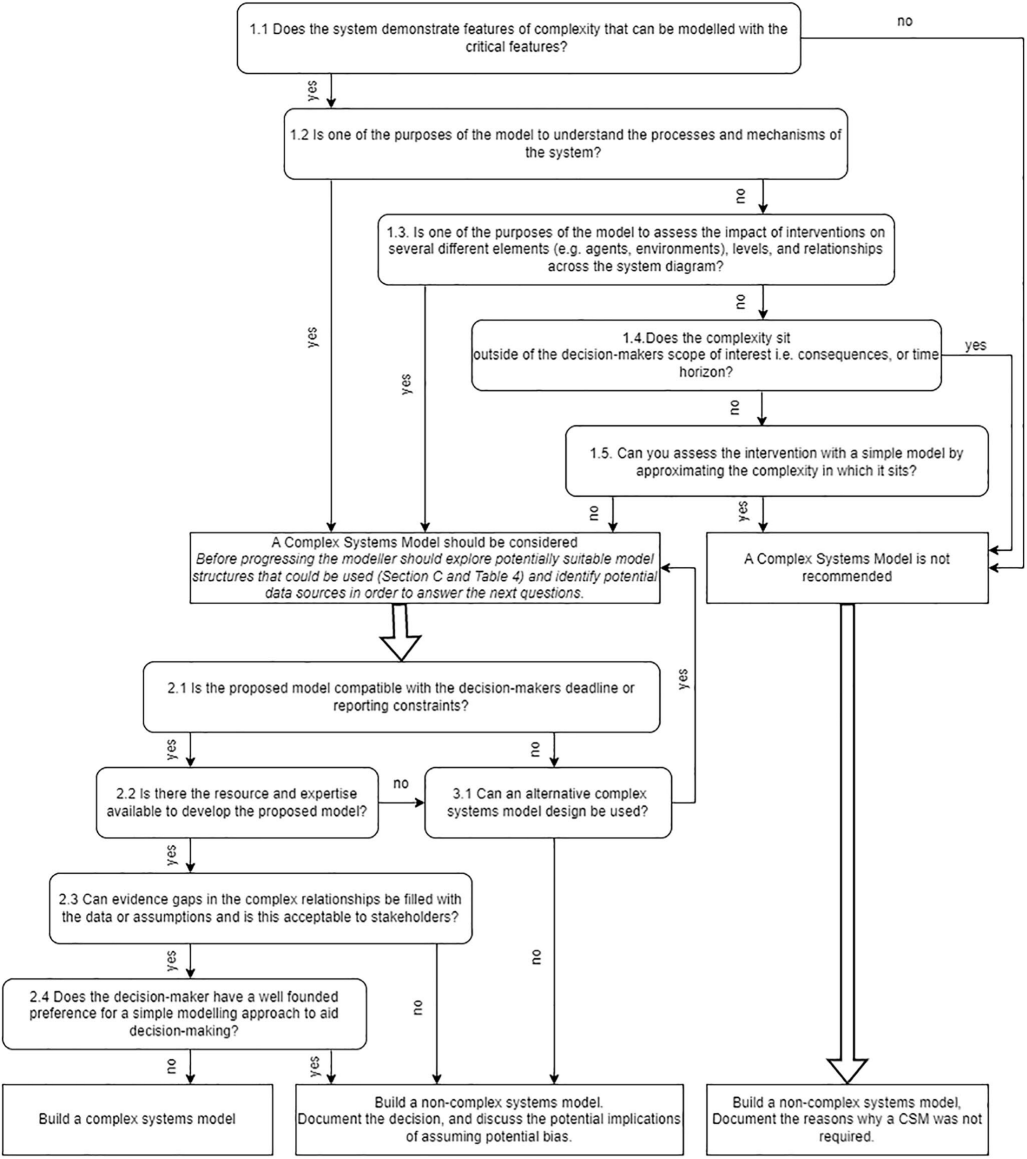
A decision tool to identify whether a complex systems model is recommended to address a decision problem.

Figure S2 in the supplementary material illustrates a framework within which the model structure is decided upon and Figure 1 provides a decision tool to help modelers identify whether to develop a CSM. The following discussion expands on the questions in Figure 1, relating them to observations from case studies, and practical considerations. Prior to using Figure 1, it would be necessary to have a detailed understanding of the system (See Section D).

Public health problems often operate in complex systems. Economic models may be developed to enhance understanding of the patterns of behavior, mechanisms and processes of a complex system, or adapted from models with this purpose. Therefore, simplification would impact the utility of the model. Probst et al. developed a CSM which aimed to implement a theoretical model of social norms in an individual‐level model to explain population‐level trends in drinking behavior (Probst et al., 2020). The interactions and feedback between individual‐level drinking behavior and social structures are inherent to the theory. They tested the theoretical framework with three hypotheses to provide insights into drinking behavior and identify interventions. In other case studies, it was possible to report whether feedback loops enhanced or mitigated intervention effects as they reverberated around the system (Keogh‐Brown et al., 2019; Occhipinti et al., 2021; Stankov et al., 2019; Tobias et al., 2010).

It is essential to understand how the intervention interacts with the system, and specifically whether the relationships linking the intervention to the consequences include the critical features of complexity. Complex interventions do not need a CSM if the effects are not expected to change the properties of a complex system (Shiell et al., 2008), or if the effects can be approximated in other ways. CSMs are particularly useful in economic evaluations of multiple interventions, applied to different elements or levels (individual or aggregate) within the model (Keogh‐Brown et al., 2019; Occhipinti et al., 2021; Probst et al., 2020; Stankov et al., 2019; Tobias et al., 2010; Viana et al., 2014). Comparisons of multiple interventions across different parts of the system are at greater risk of bias in a non‐CSM model. The effectiveness of the intervention may depend on the context in which they are applied (Dodd et al., 2010; Occhipinti et al., 2021; Viana et al., 2014), and this context may be modified by other interventions. Infectious disease models illustrate the benefits of incorporating spatial structures to characterize the context and impact interactions between individuals in the model, and transmission rates (Ferguson et al., 2006; Riley & Ferguson, 2006).

It is also important to consider what consequences, perspectives, and time horizons are important to stakeholders and/or policymakers. For example, Tobias et al. simulate the interaction of smoking with the initiation rate of smoking for future generations (Tobias et al., 2010). This feedback loop is less likely to impact the findings if future costs and QALYs are discounted.

Once the modeler has identified whether a CSM should be considered according to the issues described above it is important to consider the practical constraints of model development and these are detailed in the second half of Figure 1. It would be advisable to identify what model types might be compatible with the problem (see Section C) and what data is available to answer these questions. Model planning needs to be sufficient to consider resources, and consultation with stakeholders, and may be iterative as unexpected problems arise.

The model must be fit for purpose and developed with the decision making context in mind. Computationally expensive CSMs may limit the capacity to generate timely and comprehensive analysis for fast moving policy decisions. The modeler needs to consider where to invest time and resources to respond to the decision problem. Non‐complex models may be advantageous if their simplicity allows the modeler to accommodate other model features. The value of the investment in modeling must be balanced against opportunity costs. It is wise to keep things as simple as possible, because it can require a lot of time, effort and resources to make complexity tractable. The effort should be justified.

CSMs can require more assumptions that are not closely tied to strong experimental evidence. When developing CSMs researchers should consider how gaps in evidence will be filled and whether the tools for this are obtainable, that is, theory, data, or calibration. Combining multiple theories and adding parameters to be calibrated will make it harder to validate using external data, takes time and risks becoming less transparent and useful to stakeholders (Whitty, 2015). Increasing the number of model parameters to estimate with calibration may lead to overfitting to the data (Basu & Andrews, 2013).

It is worth considering how data gaps and the features of complexity might amplify uncertainty in the model. Calibrating multiple inputs can identify combinations of inputs that generate very different outcomes. Similarly, increasing the number of complex relationships can make the model highly sensitive to initial conditions and increase uncertainty in model outcomes. As the stimulus works its way through each set of relationships it can result in large differences in consequences (Calder et al., 2018). Additional resources may be needed to understand which relationships impact outcomes and communicate uncertainty to decision makers. Nevertheless, decisions are uncertain and simplification of the relationships risks generating incorrect and artificially precise results. Decision uncertainty is not removed by adopting a simple model, and simplification can be damaging to user confidence. Complexity will affect how the model is used and valued, so it is important to be sensitive to the needs of the decision maker in order to avoid over‐simplification or overwhelming the audience with complexity.

Non‐CSMs impose assumptions that exposure variables are independent, and relationships between exposures and outcomes are unidirectional, linear, and constant through time (Page et al., 2018). If a non‐CSM modeling approach is adopted, it is important to consider how the system violates these assumptions and be explicit about the direction of bias in the description of the model. If the features of complexity are believed to be important to the decision problem, but cannot be incorporated it will be necessary to discuss the potential limitations. Occhipinti et al. modeled suicidal behavior due to psychological distress where social determinants of psychological distress are also consequences of it (Occhipinti et al., 2021). It would be possible to have a unidirectional relationship between the social determinants and psychological distress, without feedback and under‐estimate the benefits of interventions to reduce psychological distress. By recognizing this limitation, a discussion of the direction and implications of bias in the model is possible. However, some complex relationships will be difficult to assess without incorporating the complexity in the model. For example, a similar CSM illustrated how unintended consequences for service demand negated intervention effects to produce much lower health benefits than predicted in a linear model, allowing policymakers to have strategic discussion about the whole system (Atkinson et al., 2020).

## 5 SECTIONS C: WHAT MODEL TYPES SHOULD BE USED?

### 5.1 How to select model types?

The selection of the type of model should be integrated into the decision on whether a CSM is needed with similar considerations for the decision problem, system features, and data (Figure 1). Each model type will imply different abstractions and assumptions about the system being modeled. Whilst there is a range of modeling types available, they typically differ across a few dimensions namely deterministic/non‐deterministic, static/dynamic, discrete/continuous, individual/population, mathematical logic/algebra (Calder et al., 2018). Therefore, modelers can consider the problem across these dimensions when selecting a model type. Some will accommodate certain features more easily than others.

A toolkit for model selection can be used to guide modelers to what methodology is preferred for a given problem (Jin et al., 2021). The revised Brennan toolkit includes three tools: (a) the taxonomy (Table 2), (b) the checklist (Jin et al., 2021) and (c) a decision flowchart (Squires et al., 2016). Other useful resources have been developed to map the purpose and object of the problem to system dynamics, ABM and discrete‐event simulation models simulations (Marshall et al., 2015).

**TABLE 2:** Brennan taxonomy of model structures.

			A	B	C	D
			Cohort/aggregate‐level/counts		Individual‐level	
			Expected value, continuous state, deterministic	Markovian, discreet state, stochastic	Markovian, discrete date	Non‐markovian, discrete state
1	No interaction	Untimed	Decision tree rollback or comparative risk assessment	Simulation decision tree or comparative risk assessment	Individual sampling model. Simulated patient‐level decision tree or comparative risk assessment	
2		Timed	Markov Model deterministic	Simulation Markov Model	Individual sampling model: Simulated patient‐level Markov model	
3	Interaction between entity and environment	Discrete time	System dynamics (finite difference equation)	Discrete Markov chain model	Discrete‐time individual event history model	Discrete‐time discrete event simulation
4		Continuous time	Systems dynamics (ordinary differential equations)	Continuous time Markov chain model	Continuous time individual event history model	Continuous‐time discrete event simulation
5	Interaction between heterogenous entities/spatial aspects important		x	x	x	Agent‐based simulation

*Note*: Based on the original Brennan taxonomy, and revisions (Brennan et al., 2006; A. D. Briggs et al., 2016).

### 5.2 Suggested model types for Complex System Model

System dynamics models and ABMs are commonly presented as the methods available in complex systems problems (Morshed et al., 2019). Typically, a system dynamics approach adopts an aggregate perspective, whereas ABMs allow for individual‐level simulation of behaviors, heterogeneity, and interactions between agents. However, the options for CSMs extend beyond these two dominant methods.

In other disciplines different labels may be used for model types and these are also relevant to the public health context. For example, Computable General Equilibrium (CGE) models have been used to model choices that have cross sector impacts that influence the indicators of the national economy (Keogh‐Brown et al., 2019). Partial differential equation models can be used to describe the dynamics of infectious disease transmission (Dodd et al., 2010). Both CGE and Partial differential equation models have key similarities to system dynamics and describe aggregated populations. In infectious disease modeling, the term individual‐based model is often used for ABMs. IBMs have long been used to include spatial and social population structure relevant to transmission (Riley & Ferguson, 2006), and have been commonly used in modeling COVID‐19 and impacting policy decisions (Ferguson et al., 2020). IBMs allow easy inclusion of behavioral change to project its effects on transmission dynamics (Verelst et al., 2016). Moreover, multiple approaches can be combined in what some have called Hybrid models (Brailsford et al., 2019), integrating ABM, system dynamics and discrete‐event simulation approaches into a unified model. A framework for hybrid simulation methods has been proposed (Mykoniatis & Angelopoulou, 2020), although to date very few applications have been identified within public health (Brailsford et al., 2019; Freebairn et al., 2020). We provide summary descriptions of modeling approaches in Appendix C as an introduction to the broad range of modeling options available, also providing links to relevant examples, good practice guides and web resources for further information.

Given the broad overlap among the methods described above, it may not be advisable to prescribe model types to specific public health problems, as it should depend on the nature of the question to be answered. Clusters in the adoption of model types can be driven by the research disciplines, expertise, and traditions from which they develop. Systems dynamics and CGE models are often used where the problem has broad boundaries, such as policies impacting more than public service (Keogh‐Brown et al., 2019; Occhipinti et al., 2021); ABMs tend to be used in public health where human behavior and social networks are considered important, such as understanding health behaviors around alcohol, smoking, diet and physical activity (Probst et al., 2020; Stankov et al., 2019); and discrete‐event simulations where healthcare resource constraints need to be modeled, such as cancer screening and mental health services (Brailsford et al., 2012; Viana et al., 2014). Individual level models are better at estimating the effects of heterogeneity, and exploring equity impacts.

## 6 SECTION D: WHAT PROCESSES ARE IMPORTANT WHEN DEVELOPING CSMS?

CSMs introduce additional challenges for the modeler, which can be addressed with processes, approaches, and methods described in this section and highlighted in case study examples (Table S4: supplementary material).

### 6.1 Stakeholder engagement

Strong communication with all relevant stakeholders throughout model development is essential to ensure that the model is fit for purpose (Squires et al., 2016). Within a CSM there may be broad and diverse perspectives and the choice of stakeholders may evolve during the project as the understanding of the complex system develops. Engaging stakeholders in co‐production of a CSMs has been shown to improve model transparency, understanding of the modeling process, and may build trust and acceptability of the model and its outputs (Freebairn et al., 2018). Stakeholder engagement should also include engagement with public representatives in line with recommendations for public health research (Staniszewska et al., 2021).

### 6.2 Understand and identify the problem

A documented understanding of the problem is imperative to develop and justify the model structure and type (Squires et al., 2016). The understanding of the problem will evolve as evidence becomes available, new stakeholder perspectives identified, or changes to the system occur within the timeframe of the project.

Diagrammatic representation of the system can be very useful to develop consensus and agreement between modelers and stakeholders, particularly where stakeholders have diverse perspectives. There is a vast array of approaches and methods that can be employed to develop a systems map. Commonly used methods in public health modeling include: group concept mapping (Koh et al., 2019; Lich et al., 2017), causal loop diagrams (Urwannachotima et al., 2019), and soft systems methodology (Checkland, 2000).

### 6.3 Setting the model boundary

The specification of the model boundary is somewhat subjective. The decision on the model boundary should be made transparently, justifying and documenting reasons for inclusion and exclusion of each component in the understanding of the problem (Squires et al., 2016). It is important that CSM boundaries are well described, including the level of detail for each element (Robinson, 2008). This will facilitate appropriate interpretation of the model results, considering the broader elements of the system which have not been quantified. Understanding the key variables and concepts will aid the parameterization of the model and help to prioritize where investment of resources is justified.

### 6.4 Incorporating data and evidence

Health economic models are often based on epidemiological models derived from empirical data. However, CSMs can be based on empirical observations or abstract constructions. For health economic policy evaluation CSM may use both techniques, but in most cases will require some empirical estimates and statistical techniques and causal inference. Table S4 in the supplementary material shows example statistical techniques used in our eight exemplar complex systems models. Model building and data processing require expert methodological knowledge to implement, and this can increase the expertise required for a modeling project.

#### 6.4.1 Creating synthetic individual data from observed data

Synthetic data creation techniques can be used if individual level data are not available for individual‐level models (Probst et al., 2020) and data are not available for all parts of the system. Drawing on spatial microsimulation techniques, microdata can be constructed where there is detailed attribute information available from a survey or sample dataset and sample representation from census or administrative data. Techniques such as Iterative Proportional Fitting (Lomax & Norman, 2016) can be used to reweight sample data. Alternatively, Combinatorial Optimization (Smith et al., 2021), can be used to synthesize and replicate individuals. In both cases the resulting dataset is a combination of the attribute rich (micro) data and the sample representation of the administrative (macro) data.

#### 6.4.2 Making causal inferences from observational data

Feedback loops are a critical feature of CSM, but the model parameters within feedback loops may be difficult to measure due to time‐varying confounding by other variables. A confounder is a variable that influences both the exposure and outcome variable. Time‐varying confounding occurs when confounders have values that change over time because they are also affected by the (changing) exposure (Daniel et al., 2013). Therefore, in feedback loops between exposures and confounders, time‐varying confounding is expected. In the presence of such exposure‐confounder loops, simple regression does not identify causal effects because it provides a biased estimate of the true effect of the exposure on outcome (Kuehne et al., 2022). It may be necessary to develop bespoke statistical analyses if resources and data availability allow it. Analyses should be designed alongside the conceptual mapping. A taxonomy of methods to control for time‐fixed confounding in observational studies has been developed to support statistical model selection (Ali et al., 2019). The causal analysis of empirical data affected by time‐varying confounding requires more sophisticated causal inference methods (e.g., g‐methods) (Johnson et al., 2009; Robins et al., 2004).

#### 6.4.3 Calibration of model inputs to model outputs

Calibration is the process of estimating the model parameters to obtain a match between observed and simulated patterns. A Bayesian calibration framework seeks to generate a posterior distribution of calibration parameters and model outputs, conditional on the calibration target (Chrysanthopoulou et al., 2021; Menzies et al., 2017). Non‐Bayesian methods aim to identify sets of calibration parameters for which the model best reproduces the calibration target (Chrysanthopoulou et al., 2021). Bayesian calibration has the advantage of capturing uncertainty using probability distributions, which is compatible with probabilistic sensitivity analyses (Chrysanthopoulou et al., 2021; Vanni et al., 2011), but it can be computationally demanding (Chrysanthopoulou et al., 2021). In contrast frequentist calibration methods can overfit the model by implying no uncertainty exists in the model parameters (Vanni et al., 2011). Calibration will be a necessary step in the parameterization of many CSMs, and a strength of a modeling approach, as certain parts of the model will not have data that can be used to parameterize them a priori (Occhipinti et al., 2021; Probst et al., 2020). Regardless of what calibration process is used, calibration can encounter identifiability problems where there is insufficient data for the number of parameters to calibrate (Basu & Andrews, 2013).

There are a broad range of calibration methods, although their use is often poorly documented, and non‐reproducible (Hazelbag et al., 2020). Calibration reporting guidelines have been developed (Stout et al., 2009), and should be adopted when reporting calibration.

### 6.5 Computational efficiency

Complex System Model models may encounter problems with computational capacity, particularly with micro‐level models. A computational expensive model that has many parameters to calibrate may exceed the project resource limits. Consideration of computational efficiency is important and will depend on how and when the outputs will be used. Computational limitations may require the model boundary to be revised to reduce complexity and still maintain tractability. Approaches to deal with computational efficiency, such as model emulation (Rothery et al., 2020) and distributed simulation (Taylor, 2019), could be used.

### 6.6 Model uncertainty

Sensitivity analysis and parameter uncertainty analysis can be used to communicate uncertainty to stakeholders. This can be challenging in CSMs because analyses of uncertainty may be limited by computational capacity, and other time constraints (Rothery et al., 2020), the model developers may not understand the source of uncertainty, and uncertainty can affect how answers from the model are used. Model structures, as well as parameters, may be uncertain and recent approaches have used machine learning to search across different structural configurations of a CSM (Vu et al., 2020). Proponents of a Weight of Evidence approach to evaluate and weight evidence (Dion et al., 2021) advocate holding space for multiple ways of understanding the same issue. The modeler should consider what uncertainties exist beyond what has been programmed into the model and report where complex features have not been included in the model, and how this might impact findings.

### 6.7 Model validation

Model verification and validation ensures that results from models are accurate and can be confidently used by decision makers. Model developers should consult validation typologies (Eddy et al., 2012), recommendations (Vermeer et al., 2022) and validation assessment tools, which can help assess whether sufficient validation of a CSM has been undertaken (Vemer et al., 2016). Verifying and validating a CSM is a continuous process to be performed throughout the life‐cycle of a study (Balci, 1994) and needs to be designed based on the resources and data available. CSM's may introduce additional challenges during validation, and we propose potential solutions (Table 3). It is impossible to prove that a model is valid, so verification and validation is a process of increasing confidence in a model to the point that it can be used for decision‐making (Robinson. 1999).

**TABLE 3:** Summary of challenges when undertaking validation of Public Health Complex Systems Models—categorized by ISPOR task force types of validation.

Validation type	Description	Challenges for complex systems models	Recommendations for CSM
Face validity	A review of the model structure, evidence used, and results to ensure they all make clinical and logical sense.	Challenge 1: Face validity may be more challenging to interpret due to the complexities of relationships included, meaning it is not always possible to know what results are expected given the emergent properties and feedback loops.	1) Use scenario and sensitivity analysis. 2) Identify which parts of the model contribute to outcomes or behaviors and assess whether these are plausible. Techniques developed in systems dynamics models, but applicable elsewhere, can be used to understand the contribution of feedback loops in a model and understand the origins of model behavior (Schoenberg et al., 2020) 3) Discuss results and outcomes with stakeholders to comment on whether results are consistent with expert opinion.
Verification (internal validity)	Tests the accuracy of mathematical equations and whether model structure and parameters agree with the data informing them.	Challenge 2: Complex systems computation models often involve a large amount of coding, which may make internal validity more challenging in terms of the time required.	1) Maintain up to date documentation of the code. 2) Report visual representation of model boundary to allow the code to be verified against model structure. 3) Structured walk throughs of model processes. 4) Validate inputs against their sources. 5) Independent code review 6) Double programming
	Check the model coding corresponds to the description of the model.		
Cross‐validation	The process of comparing model results with those of other models produced for the same problem.	Challenge 3: There are less likely to be other models developed to address similar public health problems to compare against.	1) Adoption of our definition of complex systems models will help to identify similar modeling approaches. 2) Greater investment is needed to fund cross‐validation of model programmes.
External validity	Compares model results with real world data.	Challenge 4: If calibration is needed the process of calibration and validation becomes connected.	A detailed description of calibration and validation processes is needed to ensure transparency in model development methods. It should be clear that the process of validation uses different data sources from the calibration process.
		Challenge 5: Real world data may not be available for all variables and consequences.	Detailed documentation of what variables and consequences have been, and have not, been validated.
Predictive validity	Compares the predicted model results to prospective real world observed events when they become available.	Challenge 6: The predictive validity of complex systems models may be difficult to establish. Real‐world shocks to the system, such as the Covid‐19 pandemic, may distort observations and change the mechanisms of the system. The feedback loops between exposure confounder and outcomes might change with time.	CSMs should be revisited and assessed for predictive validity as new data become available. The timelines and funding structures of public health projects may not facilitate this. However, public health CSMs require substantial investment and can be adapted and updated to address new public health questions. Adapting and updating models provides an opportunity for the model to develop and evolve as evidence and understanding of the system evolve.

Co‐production and model transparency can be used to ensure models are subject to face validity and verification checks, which will help build trust from decision‐makers. Examples include testing face validity and model verification through discussion of model structures with experts (Brailsford et al., 2012; Occhipinti et al., 2021; Tobias et al., 2010), Verification through discussions with stakeholders should be an iterative and multifaceted process throughout all stages of conceptual modeling, model formulation, coding and use (Williams, 2018). Model transparency should at a minimum require detailed documentation of the model to allow for it to be reproduced (Eddy et al., 2012; Vermeer et al., 2022). However, calls for “Open‐Source” models provide an opportunity to build trust in models, and improve validity (Dunlop et al., 2017).

External validation compares CSM output with retrospective data (Brailsford et al., 2012), and cross‐validation compares CSM output with other models (Viana et al., 2014). The processes of external validation and calibration may be inherently intertwined, and the stages of model calibration and validation may be indistinguishable if there are not enough data available to separate these tasks (Stankov et al., 2019). Comparing model outcomes against data not used in to inform model parameters can be challenging for public health CSM models. The data may not be routinely measured, or only measured with sampling or reporting bias. Furthermore, the feedback loops may change over time changing the relationships between exposure, confounders and outcomes. CSMs should be validated at the individual‐level, or in constituent parts, to test whether each part represents the real world with sufficient accuracy, and system‐level, or overall model, to confirm if the emergent dynamics of the system outcomes are reproduced, adding to the validation tasks required (Vermeer et al., 2022).

## 7 DISCUSSION

This paper provides a definition for CSMs and guidance on developing CSMs for public health economic modelers. It is intended as a helpful tool for economic modelers, but may also help stakeholders, those commissioning models, and those critically appraising models to identify when a CSM is justified. The definition specifies key features that distinguish complex models from non‐complex models to provide clarity to health economic modelers in the context of growing demands for complex systems approaches to public health evaluations. The absence of a clear definition of a CSM may have led to inconsistent labeling of models, less effective evidence reviews, less efficient description of methods, barriers to interdisciplinary research, and may have hampered the adoption of complex systems models.

We recommend that CSMs are required when there are processes involving dynamics, feedback loops, non‐linearity and interactions which produce emergent outcomes that matter to the decision problem. Such modeling provides a deeper understanding and analysis of the likely impact of changing factors which affect the system. We discussed useful resources to select and implement macro‐level complex system models and individual‐level models as well as hybrids of these. Finally, we identify challenges that modelers will face when modeling public health decisions and propose approaches and techniques that researchers may need to consider when designing CSMs.

Complex System Model is developing apace to support and inform public health and health policy decisions. Our hope in producing this guidance is that it can provide an accelerated learning curve, both for those new to this field and for those already involved in developing such models. We hope this document will be a lever for improved understanding and engagement with CSM and hence have an impact on public health systems policy and decision making.

This guidance document provides a step toward classifying computational models in public health according to the inclusion of complexity. The aims of this guidance document do not extend to setting research priorities for methodological development. However, workshop participants identified a lack of technical guidance specific to the task of developing CSMs for public health, meaning researchers need to access a diffuse and diverse literature from across multiple disciplines. Further research should consolidate best‐practice guidance to support skills development and training in this field. Understanding and overcoming the barriers to CSMs, for example, the resources and technical expertise needed, data requirements, limitations of model validation, will be critical to support the adoption of these methods by modelers and policymakers.

## CONFLICT OF INTEREST STATEMENT

The authors have declared funding related to their participation in this research, and have completed conflict of interest disclosure forms.

## ETHICS STATEMENT

This study did not require ethical approval.

## Supporting information

Supplementary Information S1

## Data Availability

Data sharing is not applicable to this article as no new data were created or analyzed in this study.
