# Supplementary material for: Guidance on the use of complex systems models for economic evaluations of public health interventions
Source: Health Econ. 2023 Apr 20;32(7):1603–25. doi: 10.1002/hec.4681 (PMC10947434; doi:10.1002/hec.4681)
Supplement: Supplementary file 1 — Supplementary Information S1 [file HEC-32-1603-s001.docx]

Contents

[Appendix A: Literature review 1](#_Toc130213239)

[Appendix B: Workshop 7](#_Toc130213240)

[Appendix C: Glossary of terms 9](#_Toc130213241)

[Appendix D: Tools for identifying and developing CSM 12](#_Toc130213242)

[Appendix E: Model Types 1](#_Toc130213243)

[Partial Differential Equation models 1](#_Toc130213244)

[Systems dynamics 1](#_Toc130213245)

[Computable General Equilibrium models 1](#_Toc130213246)

[Agent-based models 2](#_Toc130213247)

[Discrete-event simulation 2](#_Toc130213248)

# Appendix A: Literature review

We conducted a literature review to find examples of complex systems models in public health. The review was part of the preparations for the workshop, to develop a proposed aim and scope for the guidance. The intention was to explore the types of complex systems models that have been developed, and how they are developed, to help inform the content and structure of the workshop with the working group. Due to the exploratory nature of the review we conducted a scoping review of the literature. The aims of the scoping review were to

1. Identify case studies of complex systems models used in public health.
2. Catalogue modelling structures
3. Catalogue modelling methods
4. Catalogue data sources

We identified previously published review of complex systems methods in public health (Carey et al. 2015). The studies reported in this review and the search strategy used helped us to develop an efficient search strategy to meet the aims of this review.

*Search Strategy*

We developed a search strategy that used the studies identified in Carey et al. and supplemented it with an updated search of the literature. Carey et al. (2015) conducted a systematic review of the literature between 2000 and 2015. The review aimed to investigate the state of systems science research in public health. Within this review 36 studies were categorised as modelling studies. In order to identify more recent complex systems models we developed a search strategy to include the following search terms in Table S1. We used the search strategy from Carey et al. 2015, and adapted it to include search terms for modelling methods. The search was conducted in Medline, Pubmed and Web of Science in June 2020, but did not include studies published before Feb 2015, to avoid duplication.

*Table S1: Medline search strategy (adapted to run in Pubmed and Web of Science)*

| 1 | 'complex systems'.mp. |
| --- | --- |
| 2 | system dynamic*.mp. |
| 3 | 'systems model*'.mp. |
| 4 | 1 or 2 or 3 |
| 5 | exp Public Health/ |
| 6 | obesity/ |
| 7 | Tobacco/ |
| 8 | alcohol.mp. |
| 9 | Exercise/ |
| 10 | (social adj determinants adj2 health).mp. |
| 11 | model*.mp. |
| 12 | simulation.mp. |
| 13 | 11 or 12 |
| 14 | 6 or 7 or 8 or 9 or 10 |
| 15 | 4 and 5 and 13 and 14 |
| 16 | limit 15 to yr="2015 -Current" |

*Inclusion Criteria*

The inclusion criteria for the review were as follows.

1. A quantitative computational model
2. Addressing a public health policy problem
3. Estimates of consequences to health.
4. Feedback loops or interactions between agents.

*Data Extraction*

Data extraction included Author(s), year of publication, origin/country of origin (where the study was published or conducted), public health topic area. In addition we extracted data on the methods used in each study. We extracted summaries on the description of problem structuring/conceptual modelling, the model type (cohort vs. individual), how baseline characteristics/risk factors were described, how risk factor transitions/trajectories were parameterised, how risk functions to health outcomes were parameterised, how feedback loops were parameterised, and documented key data sources and types.

*Review Results*

*Figure S1: Flow Diagram of rapid review study identification*

Summary information for the included studies can be found in Table S2.

*Table S2: Summary details of included studies*

|  | First Author | Year | Country of Origin | Public Health topic | Model type |
| --- | --- | --- | --- | --- | --- |
| 1 | Zainal Abidin | 2014 | UK | Childhood obesity | Systems dynamics model |
| 2 | Cavana | 2008 | New Zealand | Smoking | Systems dynamics |
| 3 | Hirsch | 2010 | USA | Cardiovascular disease | Systems dynamics |
| 4 | Mahamoud | 2013 | Canada | General health | Systems dynamics |
| 5 | Metcalf | 2013 | USA | Oral Health | Hybrid model |
| 6 | Sabounchi | 2014 | USA | Obesity and fertility | Systems dynamics |
| 7 | Tawileh | 2008 | UK | Alcohol misuse | Systems dynamics |
| 8 | Tobias | 2010 | New Zealand | Smoking | Systems dynamics |
| 9 | Wakeland | 2013 | USA | Opioid use | Systems dynamics |
| 10 | Atkinson | 2018 | Australia | Alcohol harm | Agent based model |
| 11 | Brittin | 2017 | USA | Chronic disease | Systems dynamics |
| 12 | Chen | 2018 | USA | Obesity | Systems dynamics |
| 13 | Liu | 2016 | USA | Childhood obesity | Systems dynamics |
| 14 | Liu | 2018 | China | Diabetes | Systems dynamics |
| 15 | Meisel | 2016 | Colombia | Obesity | Systems dynamics |
| 16 | Orr | 2016 | USA | Obesity | Agent-based model |
| 17 | Roberts | 2019 | Australia | Childhood obesity | Systems dynamics |
| 18 | Powell | 2017 | USA | Childhood obesity | Systems dynamics |
| 19 | Shackleton | 2019 | New Zealand | Childhood obesity | Microsimulation |
| 20 | Stankov | 2019 | USA | Depression and alcohol | Agent based model |
| 21 | Urwannachotima | 2020 | Thailand | Oral health | Systems dynamics |

Our updated review highlighted that the quality and detail of reporting in complex systems models reporting remains a problem for transparency and replication. We have identified some examples of good practice where the stages of model development, structure, and data sources are explicitly described. However, the majority of studies included at least one aspect of problem structuring, initial population setup, methods for parameterisation, or outcome validation that was not described in sufficient detail for data extraction.

Cohort systems dynamics models were more commonly used than individual models. Most studies included some form of conceptual modelling; however there was often very limited detail about the process of model conceptualisation. Baseline population characteristics often require inputs from multiple sources, however studies combining numerous sources did not detail the methods used to do so. Complex systems models use a wide range of evidence and methods for model parameterisation. Most studies include more than one method or source of evidence (eg. meta-analysis and calibration) to parameterise the model. Only a few studies included a cost or economics component to their analysis.

It was particularly challenging to catalogue methods used for model parameterisation, for the following reasons:

1. In many studies the details of methods are not fully described.
2. Details of the methods may be described in referenced sources, for example regression analyses in epidemiology studies. Data extraction of secondary sources was beyond the scope of the review.
3. Some methods were described, but the methods were not easily categorised.

The limitations in reporting methods make it very difficult to identify whether robust methods have been used to develop the models. This is particularly challenging in models that have relied heavily on evidence from sources lower down the hierarchy of evidence. The lack of detail on model parameterisation would also hinder future efforts for researchers to replicate models. Methods for deriving expert opinions, calibration and assumptions are often poorly described and their limitations not discussed.

*Conclusions*

The review provided a rationale for guidance for complex systems modelling in public health. The review confirmed that very few models are developed for use in economic evaluations due to the low number that reported these outcomes. The standard of methods description and absence of model validation suggests that more guidance would be useful to establish reporting standards. The review was not useful in informing the content and key modelling methods to be included in the guidance.

Appendix Reference List: Studies included in rapid review

Atkinson JA, Prodan A, Livingston M, Knowles D, O'Donnell E, Room R*, et al.* Impacts of licensed premises trading hour policies on alcohol-related harms. *Addiction* 2018;113:1244-51.

Brittin J, Araz OM, Nam Y, Huang TTK. A system dynamics model to simulate sustainable interventions on chronic disease outcomes in an urban community. *Journal of Simulation* 2015;9:140-55.

Cavana RY, Tobias M. Integrative system dynamics: analysis of policy options for tobacco control in New Zealand. *Systems Research and Behavioral Science* 2008;25:675-94.

Chen HJ, Xue H, Liu S, Huang TTK, Wang YC, Wang Y. Obesity trend in the United States and economic intervention options to change it: A simulation study linking ecological epidemiology and system dynamics modeling. *Public Health* 2018;161:20-8.

Hirsch G, Homer J, Evans E, Zielinski A. A system dynamics model for planning cardiovascular disease interventions. *Am J Public Health* 2010;100:616-22.

Liu SY, Osgood N, Gao Q, Xue H, Wang YF. Systems simulation model for assessing the sustainability and synergistic impacts of sugar-sweetened beverages tax and revenue recycling on childhood obesity prevention. *Journal of the Operational Research Society* 2016;67:708-21.

Liu SY, Xu JC, Liu G, Xue H, Bishai D, Wang YF*, et al.* EVALUATING COST-EFFECTIVENESS OF TREATMENT OPTIONS FOR DIABETES PATIENTS USING SYSTEM DYNAMICS MODELING. In: *2018 Winter Simulation Conference*; 2018:2577-88.

Mahamoud A, Roche B, Homer J. Modelling the social determinants of health and simulating short-term and long-term intervention impacts for the city of Toronto, Canada. *Soc Sci Med* 2013;93:247-55.

Meisel JD, Sarmiento OL, Olaya C, Valdivia JA, Zarama R. A system dynamics model of the nutritional stages of the Colombian population. *Kybernetes* 2016;45:554-70.

Metcalf SS, Northridge ME, Widener MJ, Chakraborty B, Marshall SE, Lamster IB. Modeling social dimensions of oral health among older adults in urban environments. *Health Educ Behav* 2013;40:63s-73s.

Orr MG, Kaplan GA, Galea S. Neighbourhood food, physical activity, and educational environments and black/white disparities in obesity: a complex systems simulation analysis. *J Epidemiol Community Health* 2016;70:862-7.

Powell KE, Kibbe DL, Ferencik R, Soderquist C, Phillips MA, Vall EA*, et al.* Systems Thinking and Simulation Modeling to Inform Childhood Obesity Policy and Practice. *Public Health Rep* 2017;132:33s-8s.

Roberts N, Li V, Atkinson JA, Heffernan M, McDonnell G, Prodan A*, et al.* Can the Target Set for Reducing Childhood Overweight and Obesity Be Met? A System Dynamics Modelling Study in New South Wales, Australia. *Systems Research and Behavioral Science* 2019;36:36-52.

Sabounchi NS, Hovmand PS, Osgood ND, Dyck RF, Jungheim ES. A novel system dynamics model of female obesity and fertility. *Am J Public Health* 2014;104:1240-6.

Shackleton N, Chang K, Lay-Yee R, D'Souza S, Davis P, Milne B. Microsimulation model of child and adolescent overweight: making use of what we already know. *Int J Obes (Lond)* 2019;43:2322-32.

Stankov I, Yang Y, Langellier BA, Purtle J, Nelson KL, Diez Roux AV. Depression and alcohol misuse among older adults: exploring mechanisms and policy impacts using agent-based modelling. *Social Psychiatry & Psychiatric Epidemiology* 2019;54:1243-53.

Tawileh A, Almagwashi H, McIntosh S. A System Dynamics Approach to Assessing Policies to Tackle Alcohol Misuse. abstract no. 125.

Tobias MI, Cavana RY, Bloomfield A. Application of a system dynamics model to inform investment in smoking cessation services in New Zealand. *Am J Public Health* 2010;100:1274-81.

Urwannachotima N, Hanvoravongchai P, Ansah JP, Prasertsom P, Koh VRY. Impact of sugar-sweetened beverage tax on dental caries: a simulation analysis. *Bmc Oral Health* 2020;20.

Wakeland W, Nielsen A, Schmidt TD, McCarty D, Webster LR, Fitzgerald J*, et al.* Modeling the impact of simulated educational interventions on the use and abuse of pharmaceutical opioids in the United States: a report on initial efforts. *Health Educ Behav* 2013;40:74s-86s.

Zainal Abidin N, Mamat M, Dangerfield B, Zulkepli JH, Baten MA, Wibowo A. Combating Obesity through Healthy Eating Behavior: A Call for System Dynamics Optimization. *PLOS*

# Appendix B: Workshop

Workshop 1: 16th November 2020

*Session 1 Objectives: Model definitions*

1. Agree a definition of a complex system computational model

Working Definition: “A quantitative, causal model that incorporates system feedback loops, considers interactions and captures a set of important consequences of public health intervention or policy.”

2. Agree on a taxonomy of model types

Working Taxonomy from Brennan et al. (2006) ^7^.

*Session 2 Objectives: Compile a comprehensive list of existing guidance documents, case studies and methods guides.*

*Session 3 Objectives: Agree content of complex system computational model guide*

1. Develop a mind-map of key methods for complex systems modelling

2. Reach consensus on what methods should be included in the guidance document

The mind-map of key methods will be organised around the stages of model development. A working map of the stages of development is illustrated below.

Workshop 2: 17th March 2021

Session 1 Objectives: Aims and Audience

1. Refine aims and target audience of manuscript
2. Decide whether the guidance should be split into two manuscripts

Session 2 Objectives: When are complex systems models needed

1. Identify circumstances when a complex systems model is needed
2. Identify potential costs of complex systems model and circumstances where it is not beneficial.

Session 3 Objectives: Modelling types

1. Agree on how modeling types are presented and which ones to include
2. Agree main features of modelling types and illustrate in a table

Session 4 Objectives: Technical guide and case studies

1. Identify key recommendations on technical guide
2. Identify case studies for inclusion in guidance

# Appendix C: Glossary of terms

Table S3: Glossary of terms which are used in complex systems modelling disciplines

| Term/name | Description | Also known as… |
| --- | --- | --- |
| Adaptation | The elements respond dynamically to efforts to change them and do so in ways that are not predictable by looking at the relationships between elements in isolation. |  |
| Agent | A type of element which has one or more attributes and takes an active role, or decision making entity, and can produce a specified effect. In public health, ‘agents’ are often individuals, groups of people or organisations. | Individual, patient turtles |
| Attribute | A characteristic of an element. These can be static (constant over time) or dynamic (change over time). | Condition, property, state |
| Calibration | An iterative process that matches observational outcomes (calibration target) and their simulated counterparts (modelled outcomes) to inform the model parameters. |  |
| System complexity | A system that produces non-linear, adaptive and emergent outcomes. |  |
| Model complexity | A model defined to include dynamic, non-linear, feedback and interactions to describe the relationships between elements. |  |
| Consequences | The outcomes or impact to be described and captured by the model through observations of elements and their attributes over time. In order to aid public health policy-making the outcomes should include, but are not limited to, health outcomes. Outcomes describing changes to the economy, environment, social care and housing may also be of interest to public health policymakers. | Outcomes; outputs |
| Dynamic | Models that have changes in the system over time. Time can be represented in pre-specified discrete time intervals or continuous time. |  |
| Emergent Outcomes | Properties of a complex system that cannot be directly predicted from the elements within it in isolation and are more than just the sum of its parts. |  |
| Environment | In some modelling types, the environment could be characterised by a type of element to describe macro-level influences in the system, such as number of green spaces or level of air pollution. In agent-based models the environment describes the space in which agents exist. The social environment can be described as a network. An environment can be abstract (empty space), represent true geographic information, be static or dynamic. |  |
| Evaluation | Making a judgement about the value of a public health policy. This can include determining whether it can work, if it does work, does it reach those who need it, and is it worth doing in relation to other things that can be done with the resources. |  |
| Feedback | A reciprocal relationship between two elements. Feedback can be direct (A->B->A), or a chain of relationships (loop) between more than two elements within a model (A->B->C->D->E->A). | Bi-directional, simultaneity |
| Interactions | When two elements are combined, they modify the relationship to the consequence. This can occur when agents interact in an infectious disease model or when individuals interact with the environmental resource constraints to change health outcomes. Interactions can occur at a micro-level, macro-level, or between levels. |  |
| Aggregate-level model | The model represents the population as aggregated groups formed by combining individuals or representing institutions or sectors or norms. The characteristics of the aggregated group are reflected through population averages, rather than heterogenous and divisible. | cohort; population-level Macro-level |
| Individual-level model | The model simulates multiple individuals as single elements that cannot be divided. Therefore, the heterogeneity and characteristics within the population can be more completely reflected. | agent-based, Micro-level |
| Model Boundary | A description of what elements and relationships will be included in the computational model (and what is excluded). The understanding of the problem will be broader than the model boundary 5. |  |
| Non-linearity | A change to an element leads to a non-proportional change to another. Non-linearity can be programmed directly into the relationships between model elements. Alternatively, it may emerge from the feedback and interactions within the model. |  |
| Population | A type of element used to describe an aggregate-level grouping, such as a group of individuals, with one or more attributes. | Cohort |
| Relationships | The way that elements connect to other elements in the model. Connections can imply directional causality, such that changes to one lead to changes in the other, can be derived from other elements, or represent feedback such that the direction of effect works both ways. These links can be visually described by arrows in a conceptual diagram. The relationships included in the model boundary need to be quantified in some way in a computational model. When quantified, the relationship is usually a mathematical function/formula that sets out how one element changes the value or attributes of another element over time. | Interconnections |
| Sectors | Organisational sub-structures or components within the system. |  |
| Systems map | A visual representation of the system depicting the relationships between elements using symbols, such as arrows, to indicate direction of causation or association that can be easily understood. | Conceptual map, logic model, mind map, causal loop diagram. |
| Threshold effects | Discontinuities where a response only occurs when an element, or multiple elements, reach a critical value. | Tipping point  Ceiling effect |
| Validation | Testing how accurately a model reflects what happens in the real world. |  |
| Verification | A process to check if the model is coded correctly. |  |

# Appendix D: Tools for identifying and developing CSM

Figure S1: A visual description of the critical features of complexity in CSMs


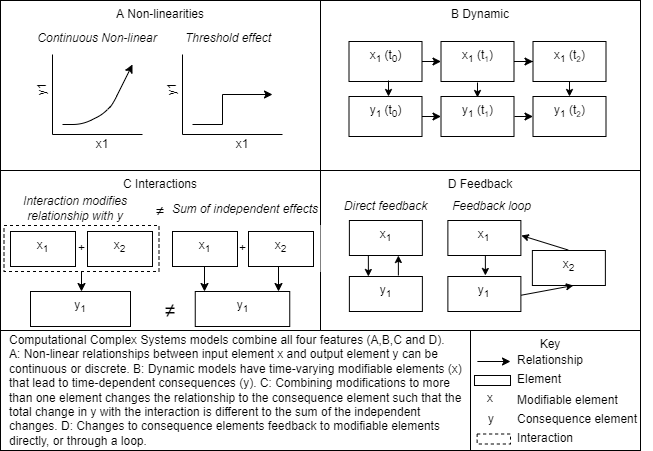


Figure S2: Overview of a modelling framework for public health economic models.


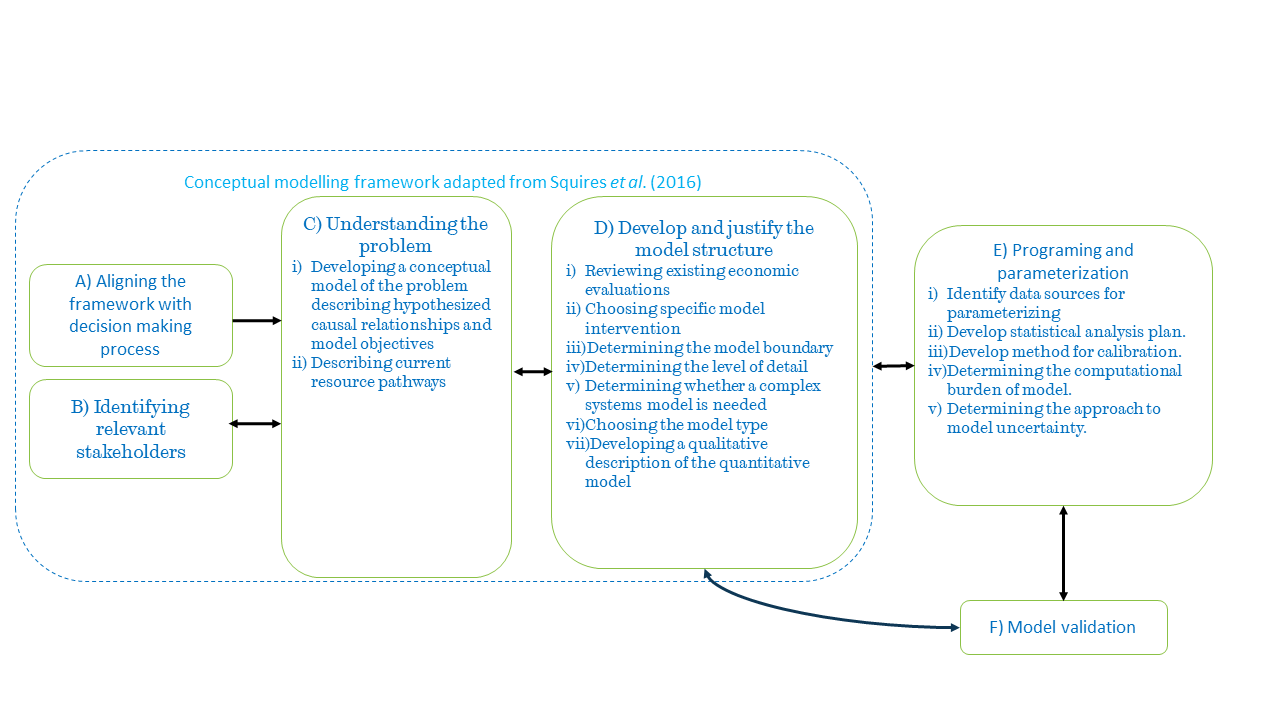


Table S4: Methodological, statistical and computational approaches used in the eight selected Public Health CSM case studies

| Authors | Model type | Software | Conceptual Modelling | Statistical Techniques/model parameterisation | Model Validation |
| --- | --- | --- | --- | --- | --- |
| Dodd et al. 2010 | Partial Differential Equations | R, Python | Literature was used to inform the dynamics of infectiousness and impact of ART; exemplar parameters for R0 in different risk groups, their relative size, and HIV infection incidence were chosen by subject expert co-authors. | Infectiousness over time and ART effect in reducing infectiousness were based on published analyses of cohort studies. Calibration to equilibrium incidence was achieved by numerically solving for the risk heterogeneity parameter. | An iterative numerical approach to solving for equilibrium was developed and validated against a numerical solution of the PDE dynamics. |
| Brailsford et al. 2012 | Discrete-Event Simulation | Microsoft Visual Basic 6.0 | The model made use of survey data collected on the psychological constructs associated with the theory of health behaviour and attendance at breast cancer screening. From this the researchers developed a model to determine attendance conditional on psychological constructs. | The study adopted four models of tumour growth (exponential, Gompertz, modified Gompertz, and logistic) to allow for different assumptions regarding the rate of tumour growth. Each assumption is presented to allow the sensitivity of the model to the assumption to be explored. | Tests of face validity and operational validity are described. The model assumptions were discussed with experts. Model outputs were compared with national statistics. |
| Probst et al. 2020 | Agent Based Models | C++ using the Repast HPC agent-based modelling platform | The conceptual model was developed by translating theoretical concepts into behavioral rules and mechanisms. | A sociodemographic microsynthesis was used to populate the individuals with characteristics of age, gender, and drinking history in the model. The microsynthesis was representative of the population of the United States in 1979 and contained individuals aged 12 to 80 years informed by three datasets.  Model parameters were calibrated using Bayesian principles based on prior distributions. Parameter sets were sampled from the joint prior distribution using a Latin hypercube space-filling design. The model was run for each parameter set and the fitness was calculated by comparing the simulated outputs and the empirical target data. | No explicit mention of validation. However, the extensive calibration tasks used to develop the model ensure that the model reproduce historical data. |
| Tobias et al. (2010) | System Dynamics | iThink software | Researchers created a user interface for the model where key model parameters can be adjusted by clinicians, public health experts, or policymakers, thus creating their own ‘what-if’ scenarios. This knowledge mobilisation technique increases user understanding of the model and its results, and is a useful tool for policy design. | Researchers derived input parameters such as smoking initiation/ quit rates, quitting effectiveness, smoking intensity, the effect of peer and parent feedback on smoking initiation, and mortality hazard ratios from several data sources including a Cochrane review, a census, and a tobacco use survey.  The model was calibrated by ensuring that it could replicate smoking prevalence and mortality by age, resulting in minor adjustments of input parameters. | Population projections were compared with other national forecasts. Tests of face validity were also conducted, and the structure and output of the model were discussed with subject-matter experts. |
| Occhipinti, A et al. 2021 | System Dynamics | Stella Architect ver. 1.9.4 (www.iseesystems. com) | The researchers adopted a Participatory Modelling Approach. 50 local stakeholders were engaged through a series of workshops, meetings, priority setting surveys and system mapping activities conducted in 2019. Stakeholders were provided with the opportunity to interact directly with the model interface to run scenarios, test alternative assumptions, discuss and question results, and provide feedback on interface design and functionality. | The researchers used constrained optimisation to estimate parameters that could not be estimated from the literature. The parameters were calibrated using time series data for multiple outputs to obtain the set of (optimal) parameter values minimising the sum of the mean absolute percent error calculated for each time series separately. | Tests of face validity were also conducted, and the structure and output of the model were discussed with subject-matter experts.  Model outputs were compared against retrospective time series data across a range of indicators including prevalence of psychological distress, mental health-related emergency department presentations, psychiatric hospitalisations, self-harm hospitalisations and suicide deaths (for youth and total population). |
| Keough-Brown et al. 2019 | Computable General Equilibrium | food systems | The model adopted and extended an existing CGE model and therefore the majority of the modelling framework was already specified. A conceptual map to describe the relationships between the model of the economy and the health sub-model is provided. | The CGE model was calibrated to Thailand specific data. The calibration technique was not described.  Other model parameters were informed by published literature. | CGE is a counterfactual model such that scenarios are compared with a baseline - one can add multiple scenarios, but there are not traditional validation techniques as usually seen in micro models. |
| Stankov et al. (2019) | Agent Based Models | Netlogo v5.3.1 | A causal loop diagram depicts the  interrelationships between excessive alcohol consumption and depression, including factors hypothesised to influence their co-evolution. | To test the plausibility of the underlying model structure, the researchers used pattern-oriented modelling, which involves examining whether the model can reproduce ‘patterns’ that are observed in the real-world system. In this study this involved using the prevalence of depression and alcohol misuse found in data from older adults in Los Angeles county to calibrate the parameters of the model.  Model calibration was required to determine parameter values for the relative influence of individual, social and environmental factors on drinking behaviour and depression. When model calibration provided multiple plausible configurations of the model parameters, results from the literature on the impact of these factors to select options. Namely, higher weights were assigned to individual level influences due to more consistent effects being found for these factors in the literature. | No validation tests were described beyond those discussed in the calibration column. |
| Viana et al. 2014 | Hybrid Models | VenSim, Microsoft Visual Basic, Simul8. | The SD model was adapted from an existing model to describe Chlamydia screening. The DES model was developed with clinic staff and key decision variables were set in collaboration with clinic staff | The SD model was developed in VENSIM and the DES in Simul8. The models were programmed to transfer data using excel, utilising Visual Basic. | The SD model was validated and tested by comparing results with existing SD models of Chlamydia infections. |

# Appendix E: Model Types

## Partial Differential Equation models

Partial differential equations (PDEs) are most commonly used in modelling relevant to health for describing multiple time-scales. For example, PDEs can be used to model system dynamics alongside aging, with age being a second time variable that advances at the same rate as calendar time. Other applications of this type include measuring dependence on the time since entering a state. Examples of this type of application include capturing varying infectiousness by time-since-infection, or time-varying hazards of progression between states. PDEs can also be used to describe the dynamics of systems that extend in space, including diffusive or wave-like behaviour. Non-linear PDEs have long been studied as model descriptions of continuous physical systems, such as the Navier-Stokes equations of fluid dynamics, which give rise to emergent behaviour such as turbulence. PDEs, in common with ordinary differential equations, describe aggregated populations and assume average behaviours apply within these coarse-grained states. However, the presence of continuously varying state-indices (eg age) can allow inclusion of finely graduated heterogeneity that depends on these indices.

## Systems dynamics

System dynamics (SD) is a methodology which explicitly enables highlighting the existence of feedback loops, time delays, and nonlinearities in a system. One of its basic principles is that the structure of the system is the one responsible for driving the system’s behaviour (Forrester, 1992; Sterman, 2000). This modelling method is typically used to analyse problems from an aggregate perspective and enables a more holistic understanding of the structures behind a complex phenomenon. SD starts with the definition of a problem (e.g. system mapping by incorporating different knowledge sources (Vennix, 1996) and then moves on to develop a computational model of the causal relationships between the system elements in order to find changes (e.g. leverage points) in the system structure and policies to address the issue (Forrester, 1992).

SD modelling can be done qualitatively, using Causal Loop Diagrams (CLDs), and quantitatively, by implementing quantified models with equations to simulate how a system might behave over time given a set of conditions and assumptions. These two approaches complement each other.

System dynamic modelling allows researchers to change values in parts of the model to simulate the impacts and unfolding effects of interventions in different contexts and within a wider complex system. Martinez-Moyano and Richardson (2013) provide a best practice guide for developing system dynamics models.

## Computable General Equilibrium models

Computable General Equilibrium models (macroeconomic models) are concerned with choices and activities that cross the markets (or ‘sectors’) that comprise an ‘economy’ – typically this space is defined in geographical terms, principally a ‘national’ economy (Smith et al. 2014; Smith et al. 2015; Smith et al. 2018). The system level here is the ‘sector’, which covers everything from health care and education to manufacturing and services, and sub-divisions to for example pharmaceuticals, washing machines or restaurants, and also incorporate imports and exports. Indicators range from top-level Gross Domestic Product (a measure of whole economy ‘wealth’) to inflation, employment, tax income and balance-of-payments; many of which may be disaggregated further into each sector of interest. Given its level of operation, data for macro-models is typically from national statistics, with scenarios to be modelled integrating micro-based data. Macro-economic models can cover very broad, crude, models such as growth models, where health is one independent variable impacting growth of GDP for example, through to micro-based whole-economy models such as Dynamic Computable General Equilibrium (DCGE) models, where the development of the economy over discrete time periods (typically annual) is established and then subject to counter-factual analysis based on policies or shocks of interest which typically comprise stochastic scenarios. Interactions within the DCGE model are determined by a set of elasticities across price and income that govern the relationships between the sectors of interest; such that a change in price of, say, petrol will affect multiple sectors in terms of their prices, affecting supply and demand across sectors, and feedback in to the subsequent period price of petrol etc, until a new equilibrium position is found. As these changes occur they have implications for health (eg changes in diet, exercise, health care provision) and the health changes also impact the economy (typically through changes in productivity associated with changes in mortality and morbidity).

## Agent-based models

Agent-based Models (ABMs) simulate interacting autonomous agents (e.g., individuals, households) situated in space and time. The interplay of individual differences and interactions between agents allow us to identify causal mechanisms which generate emergent social phenomena (Bianchi et al. 2015). Agents are assigned attributes, capacities, and resources. Rules are implemented to govern actions and interactions; these can be simple heuristics or defined by theory. Generally, ABMs have two system levels, the micro or “agent level” consisting of agents or the collective actions of agents, and the macro level consisting of the broader social and structural entities. The Mechanism Based Social System Modelling architecture describes four mechanisms which can be used to specify interactions between these levels: Situational mechanisms (the impacts of social structures on individuals’ internal states), action mechanisms (the impact of internal states on agent action/behaviour), transformational mechanisms (how the actions of agents then shape structural entities) and macro-macro mechanisms (how social entities directly affect other social entities) (Vu et al. 2020). ABMs can incorporate feedback loops, non-linearity, and a multiplicity of factors. Individual actions and interactions in these models are to an extent the outcome of decision making by agents, however there is also an element of stochasticity as they are often determined by probability distributions. Gilbert (2019) provide a guide to agent-based models.

## Discrete-event simulation

A discrete event simulation (DES), otherwise known as a time-to-event simulation, is commonly used to characterise and analyse systems in which resources are shared between agents (Law, 2007). A DES is run in continuous time, with entities experiencing their next event at a time determined by a random draw from the time-to-event distributions for each possible event. In the context of health, agents tend to be individuals with heterogeneous characteristics which can change over time, because of previous events or external forces (Zhang, 2018). DES events can be determined by time-invariant or time-dependent characteristics based on mathematical function describing the relationship.

DES models are typically used in models in which agents compete for resources, such as a hospital emergency department, since they can incorporate the availability of resources (e.g. ICU beds) in continuous time (Marshall et al. 2015; Salleh et al. 2017). While in some cases, for example where times to event are irregular and widely distributed, DES models can be more computationally efficient and accurate than STMs, in most cases DES models are particularly computationally burdensome (Degeling et al. 2018; Standfield et al. 2017). They are also particularly data-intensive since the time distribution between each set of events is required to determine how an agent progresses through the model. Karnon provide a guide to discrete event simulation models.

Network Science

Network Science (now a broad multi-disciplinary field in itself) has evolved suites of tools to help us understand the networked world that we live in and to shed light on many of the complex public health problems that we face today. Their focus on the relationships between elements of a complex system and the influences acting between them, at different levels, has given them traction on a wide range of public health issues, for example, helping us understand “contagion” processes both in communicable disease control and in the cultural and political influences on chronic disease (Valente and Pitts, 2017). So while one might consider the network science approach to be more focussed around mechanisms of change within a complex system, choosing to adopt a network perspective and the modelling tools that it implies, will dictate the type of data (on, for example, network position, structure, modularity and centrality) that a modeller will need in order to exploit its potential and this needs to be borne in mind when designing any ex ante system evaluation or computational model.

Systems Engineering

Systems Engineering (CSE) share this focus on mechanisms of influence and aims to help us understand how a dynamical system can be regulated to achieve a preferred outcome and the idiographic, time-varying and non-linear nature of human behaviour is increasingly being studied using CSE principles (Phatak et al. 2018). This is understandable given the much greater acknowledgment of the need for public health intervention to be adaptive.

Case Based Scenario Simulation

Machine Learning and AI based methods, grounded on the principles of case based reasoning, such as Case Based Scenario Simulation (CBSS) offer further potential in public health modelling (Schimpf et al. 2021). Drawing on methods of cluster and configurational analysis CBSS aims to bridge the computational/quantitative/qualitative divide and is not so much about identifying some underlying causal model, as it is about exploring how various interventions might unfold for a given policy and the larger complex system in which it is situated.

Hybrid Models

It may also be appropriate to fuse multiple approaches, explaining the emergence of what some have call Hybrid models (Mykoniatis and Angelopoulou, 2020). With potential yet to be fully realised in addressing of complex public health problems, a MPSM approach at least would force the modeller to let the particular problem and the data, rather than a particular bespoke tool, drive the choice of most appropriate analysis. Multi-paradigm software packages such as ANYLOGIC (www.xjtek.com/AnyLogic) can facilitate hybrid model. However, it is also possible to programme hybrid models from scratch, or keep models in separate software packages, but allow them to communicate across other software (See Viana et al. example).

Reference List

Bianchi F, Squazzoni F. Agent-based models in sociology. WIREs Comput Stat. 2015;7:284–306.

Carey G, Malbon E, Carey N, Joyce A, Crammond B, Carey A. Systems science and systems thinking for public health: a systematic review of the field. BMJ open. 2015 Dec 1;5(12):e009002.

Degeling, K., Franken, M.D., May, A.M., van Oijen, M.G., Koopman, M., Punt, C.J., IJzerman, M.J. and Koffijberg, H., 2018. Matching the model with the evidence: comparing discrete event simulation and state-transition modeling for time-to-event predictions in a cost-effectiveness analysis of treatment in metastatic colorectal cancer patients. *Cancer epidemiology*, *57*, pp.60-67.

Forrester, J. W. (1992) “Policies, Decisions and Information Sources for Modeling,” *European Journal of Operational Research*, 59(1), pp. 42–63. doi: 10.1016/0377-2217(92)90006-U.

Gilbert, N. (2019). Agent-based models (2nd ed.). Sage Publications Inc.

Karnon J, Stahl JE, Brennan A, et al. Modeling using discrete event simulation: a report of the ISPOR-SMDM Modeling Good Research Practices Task Force-4. Value Health. 2012;15(5):821-827.

Law A.M., Simulation Modeling and Analysis, McGraw-Hill Higher Education, 2007.

Marshall DA et al ISPOR Emerging Good Practices Task Force. Selecting a dynamic simulation modeling method for health care delivery research – Part 2. Value in Health. March 2015b 18(2): 147-160.

Martinez‐Moyano, I.J. and Richardson, G.P. (2013), Best practices in system dynamics modeling. Syst. Dyn. Rev., 29: 102-123. <https://doi.org/10.1002/sdr.1495>

Mykoniatis K, Angelopoulou A. A modeling framework for the application of multi-paradigm simulation methods. Simulation. 2020 Jan;96(1):55-73.

Phatak SS, Freigoun MT, Martín CA, Rivera DE, Korinek EV, Adams MA, Buman MP, Klasnja P, Hekler EB. Modeling individual differences: A case study of the application of system identification for personalizing a physical activity intervention. Journal of biomedical informatics. 2018 Mar 1;79:82-97.

Salleh, S., Thokala, P., Brennan, A., Hughes, R. and Dixon, S., 2017. Discrete event simulation-based resource modelling in health technology assessment. *Pharmacoeconomics*, *35*(10), pp.989-1006.

Schimpf C, Barbrook-Johnson P, Castellani B. Cased-based modelling and scenario simulation for ex-post evaluation. Evaluation. 2021 Jan;27(1):116-37.

Smith RD. Health and Health Care, Macroeconomics of. In: Anthony J. Culyer (ed.), *Encyclopedia of Health Economics*, Vol 1. San Diego: Elsevier; 2014. pp. 327-332

Smith RD, Keogh-Brown M, Hanefeld J. Macroeconomics, Trade and Health. In: Scheffler, RM (ed.), *World Scientific Handbook of Global Health Economics and Public Policy*, World Scientific; 2015. Volume 1; Chapter 2.

Smith RD, Prah Ruger J, Jamison DT, Bloom DE, Canning D. *Health and the Economy*. In: Merson M, Black R, Mills A (eds), “Global Health: diseases, programs, systems and policies”, Jones & Bartlett Learning, LLC; 2018. Chapter 19: 835-855.

Standfield, L.B., Comans, T.A. and Scuffham, P.A., 2017. An empirical comparison of Markov cohort modeling and discrete event simulation in a capacity-constrained health care setting. *The European Journal of Health Economics*, *18*(1), pp.33-47.

Sterman, J. (2000) *Business dynamics : systems thinking and modeling for a complex world*. Boston: Irwin/McGraw-Hill.

Valente TW, Pitts SR. An appraisal of social network theory and analysis as applied to public health: challenges and opportunities. Annual review of public health. 2017 Mar 20;38:103-18.

Vennix, J. A. M. (1996) *Group model building : facilitating team learning using system dynamics*. Chichester: J. Wiley.

Vu TM, Probst C, Nielsen A, Bai H, Buckley C, Meier PS, et al. A software architecture for mechanism- based social systems modelling in agent- based simulation models. JASS. 2020;

Zhang, X., 2018. Application of discrete event simulation in health care: a systematic review. *BMC health services research*, *18*(1), pp.1-11.
